# Challenges achieving horizontal coherence across health and public security policies in formulating Uruguay’s cannabis regulation

**DOI:** 10.1093/heapro/daae136

**Published:** 2024-11-04

**Authors:** Rachel Ann Barry

**Affiliations:** Tobacco Control Research Group, University of Bath, Claverton Down, Bath, BA2 7AY, UK; Global Health Policy Unit, University of Edinburgh, Old College, South Bridge, Edinburgh, EH8 9YL, UK; SPECTRUM Consortium, Usher Institute, Old Medical School, Edinburgh, EH8 9AG, UK

**Keywords:** cannabis regulation, policy coherence, unhealthy commodity regulation, policy formulation, Uruguay

## Abstract

In 2013, Uruguay became the first country to regulate the legal production, distribution and sale of recreational cannabis. While key officials have framed Uruguay’s landmark legislation as part of the government’s strategy to regulate cannabis, tobacco and alcohol, there is limited empirical research exploring the political considerations that influenced its approach. Drawing on the concept of policy coherence—the process by which policymakers seek to minimize conflicts and maximize synergies across policy agendas—this study explores the extent to which Uruguay’s cannabis regulation was influenced by the promotion of policy coherence within health and across other policy spheres. Government documents, 43 semi-structured interviews and field observations were thematically analysed. The analysis shows that the pursuit of policy coherence across health issues was relatively limited, and where there is an element of regulatory coherence, there also appears to be minimal coordination. Efforts to promote substantive policy coherence were shaped by a desire to legitimate cannabis use without creating an upstream driver or structural force that would promote excessive consumption. The findings also reveal that the outcome of Uruguay’s cannabis regulation was more directly shaped by broader political considerations, including how to resolve tensions between public security and unhealthy commodity regulation goals. This study raises important questions around the extent to which Uruguay’s cannabis regulation was shaped by the explicit goal of policy coherence, suggesting rather that comparisons with tobacco and alcohol regulation were strategically used to justify the introduction of a legally regulated cannabis market.

**Contribution to Health Promotion**

- This study identifies how the pursuit of policy coherence across cannabis, tobacco and alcohol was challenged by a neoliberal ideology underlying Uruguay’s public security strategy of cannabis regulation.
- Public health advocates should be cautioned by the synergies that such neoliberal ideas create with relevant economic spheres that have a bearing on health policy, particularly as more jurisdictions liberalize recreational cannabis supply.
- These findings raise questions around whether policymakers should treat policy coherence as a normative goal, suggesting rather that the pursuit of policy coherence may inhibit a more nuanced (and potentially more effective) approach to regulating cross-cutting issues.

## BACKGROUND

In 2013, Uruguay became the first country to regulate the legal production, distribution and sale of recreational cannabis. Uruguay’s regulatory approach provides legal access to cannabis through multiple supply channels, including home cultivation, cannabis clubs and commercial sales through pharmacies (Queirolo *et al*., 2019; Barry, 2023). Officials involved in developing Uruguay’s landmark legislation have framed it as part of the government’s strategy to regulate cannabis, tobacco and alcohol, reflecting the state’s wider commitments to public health and rights-based approaches (Cánepa, 2013; Romani, 2015; Roballo, 2017). While several studies show prima facie evidence of policy similarities between Uruguay’s tobacco (Arocena and Aguiar, 2017; Forné, 2017; Musto, 2018; Rivera-Vélez, 2018) and alcohol approaches (Arocena and Aguiar, 2017; Forné, 2017; Musto, 2018; Rivera-Vélez, 2018), there is little empirical research exploring the processes by which knowledge, practices and policy tools from these experiences informed the development of cannabis regulation. Moreover, other research suggests that policymakers promoted the reform in response to a rise in public insecurity triggered by a series of violent robberies and other drug-related crimes (Repetto, 2014; Queirolo *et al*., 2019), raising questions around the extent to which development of Uruguay’s cannabis regulation was shaped or constrained by potentially competing policy agendas beyond health.

This article aims to address this gap by exploring the extent to which Uruguay’s cannabis regulation was influenced by the pursuit of policy coherence within health and across other policy spheres. For the purposes of this analysis, policy coherence may be seen as the ‘extent to which conflicts between policy agendas are minimized and synergies maximized’ (Blouin, 2007). Developing a coherent approach to regulating the commercial determinants of health (CDoH) or the ‘systems, practices, and pathways through which commercial actors drive health and equity’ (Gilmore *et al*., 2023), has been a significant challenge at both global and domestic levels (Collin and Hill, 2015; Hawkins *et al*., 2018), reflecting the different objectives of various state sectors, including trade, agriculture and health (Lencucha and Thow, 2019). Moreover, in Uruguay and globally, cannabis remains largely outside of broader governance debates around regulating the CDoH, raising questions around the ways in which a rhetorical commitment to policy coherence was pursued as a normative goal of cannabis regulation in Uruguay.

In the context of the CDoH, health advocates and researchers tend to treat all unhealthy commodity producers (UCPs), for example, tobacco, alcohol, gambling, the same, assuming that policy coherence in health governance is then approached in similar ways (Lencucha and Thow, 2020). However, this assumption inhibits a more nuanced understanding of how different product categories and industries are regulated across diverse contexts (Hawkins *et al*., 2018) such that the same broad coherence approach might produce divergent policy outcomes in different settings. Greater attention must also be paid to the challenges of coherence (Barry *et al*., 2022) and the underlying drivers of regulation in terms of framing, social context and institutional environment (Lencucha and Thow, 2019). This study seeks to address these vital knowledge gaps by analysing the conditions that foster policy (in)coherence across health and other policies in the context of regulating a nascent industry.

In the following sections, the conceptual framework is presented outlining the ways in which coherence is operationalized in this study, after which the mixed-methods design and approach to data analysis are described. Next, the findings are presented, and the article concludes with their potential implications for health governance and policy coherence.

## ANALYTICAL APPROACH

**Fig. 1:**
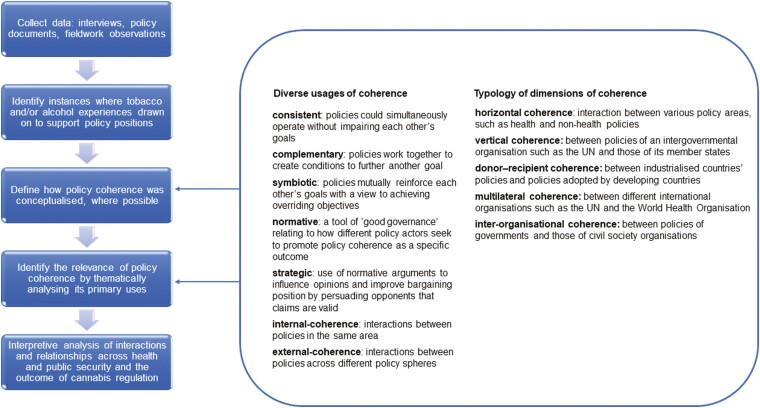
Conceptual framework of policy coherence and its diverse dimensions.

The analytical approach was informed by previous studies of the challenges to achieve policy coherence across health and trade agendas within the global health policy literature (Blouin, 2007; Collin, 2012; Battams and Townsend, 2018). This involved thematic analysis of the ways the pursuit of policy coherence influenced the development of Uruguay’s cannabis regulation to assess and interpret whether interactions between health and other policies were viewed as mutually reinforcing (symbiotic), complementary or at least not neutralizing each other’s goals (consistent). This interpretive lens is based on an understanding of policy coherence not as an absolute or fixed term (Hommels *et al*., 2013) but rather one that can have different meanings according to the priorities and goals of those promoting it as a strategy (Carbone, 2008). Figure 1 provides an overview of the analytical approach including the conceptual framework of policy coherence and its diverse dimensions used to thematically code the data.

This study focuses on two dimensions of policy coherence within government. Cannabis regulation is a complex, multidimensional issue that intersects across diverse policy spheres and governance levels. Regulation of the cannabis market therefore requires involvement of government departments and agencies beyond health, including public security, justice, agriculture, finance and education (Muscat and Pike, 2012). The importance of multisectoral dialogue and decision-making on cannabis regulation also points towards potential challenges around the development of policy that can effectively manage the interactions across the diverse goals and priorities of relevant stakeholders involved, including by reducing conflicts and minimizing trade-offs (Nilsson *et al*., 2012). In this context, it is important to consider the ways in which other policies and objectives interact with the health goals of cannabis regulation: how these goals may be neutralized or even impaired by the impact of other policies.

The study draws on the typology of diverse dimensions of coherence suggested by Siitonsen (2016) to explore the extent to which policy actors sought to align practices, policies and objectives in the formulation of Uruguay’s cannabis regulation. Specifically, it focuses on horizontal coherence, which involves interactions between various policy areas and is distinguished from vertical coherence, which involves interactions between policies across diverse governance levels (Siitonsen, 2016). Horizontal coherence can be both internal and external. Internal-horizontal coherence refers to interactions between policies within the same area, whereas external-horizontal coherence refers to interactions across different policy spheres.

In the context of Uruguay, cannabis regulation has been presented as strongly linked to the state’s historic and contemporary approaches to tobacco (Spithoff *et al*., 2015; Hughes, 2018) and alcohol control (Arocena and Aguiar, 2017; Forné, 2017; Musto, 2018; Rivera-Vélez, 2018). This dimension is treated as internal because it explores the consistency in governance approaches across cannabis health policy and that of unhealthy commodity regulation, with reference to tobacco and alcohol. The question of the relevance and impacts of other policies on health objectives is prompted by the observation that policy paradigms are difficult to change (Hall, 1993) and the health goals of cannabis regulation may be linked with or even subordinated to other political objectives or interests (Ashoff, 2005). In this case, the analysis explores the consistency in governance approaches between Uruguay’s unhealthy commodity regulation policy and that of public security.

In this study, horizontal coherence is operationalized in terms of the different policies that influence the supply and demand for psychoactive substances. As a normative goal, policy coherence is achieved where such policies reinforce or at the very least do not neutralize or impair each other’s goals. Conversely, policy incoherence can also refer to the negative interactions of policies across different spheres (Collin, 2012), which either counteract or may even cancel out the effects of one another in the pursuit of different overriding objectives. In this case, policy inconsistencies are discussed with reference to how public security policies are seen to influence cannabis regulation in ways inconsistent with Uruguay’s unhealthy commodity regulation policy at the population level.

### Methods

This article is part of a larger project exploring whether development of Uruguay’s cannabis regulation could be understood with reference to the pursuit of policy coherence in health governance across horizontal and vertical dimensions (Barry, 2023). This exploratory research adopted a qualitative, intrinsic case study design used to gain critical insights into an emerging phenomenon with unusual interest and needs to be described in detail (Grandy, 2009). The methodology reflects a mixed-methods approach using a combination of semi-structured interviews with key policy actors, conducted in accordance with the University of Edinburgh’s ethics committee, documentary analysis and fieldwork observations.

### Interviews

Interviewees were provided an information sheet in advance of the interview and requested to provide informed consent to participate in the study, to be audio recorded and indicate how their data could be used and attributed to them. This is consistent with standard methodological and ethical procedures for research involving human participants (Stark and Hedgecoe, 2010). Interviews lasted between 30 min and 4 h (with most around 60 min).

Interview requests were sent to 71 potential participants. Access was gained to initial participants by drawing on informal relationships the researcher had built with three key people or ‘gatekeepers’ from health and drug policy sectors in Uruguay. Two were approached via email in June 2017 requesting their participation in the research and one provided a list of potential interviewees (Supplementary Appendix A). Additional participants were identified through analysis of policy documents (purposive sampling) and from suggestions of interviewees (snowball sampling). Policy actors were eligible for inclusion if they met two criteria: (i) were actively engaged in the development of Uruguay’s cannabis regulation; or (ii) had participated on behalf of an organization or state entity in this process. Data collection continued until additional interviewees and documents no longer provided new perspectives or insights into the relevance of the pursuit of horizontal coherence in shaping the development of Uruguay’s cannabis regulation in ways that would significantly enhance the findings.

A total of 43 interviews were conducted with key actors, including policymakers, legal and medical experts, health and drug advocates, international NGOs and the commercial sector (see Table 1). Of these, 39 were conducted in Spanish and four in English, two were paired and four carried out online using Skype, owing to the geographical and economic challenges of attempting to conduct face-to-face interviews with international experts living outside of Uruguay. All interviews contained material relevant to the study and were transcribed verbatim either by RB (*n* = 38) or a professional transcriber (*n* = 5), the latter of which due to their length. The project aimed to interview a diverse range of policy actors advocating different positions on the problem of cannabis and its potential solutions. Emphasis on diverse perspectives on cannabis regulation reflects the wider focus of the study from which this article is taken, but they also provided important insights about the potential implications of competing priorities for the pursuit of policy coherence in health governance.

**Table 1:** Interview participant characteristics (*N* = 43)

Geographic remit	Policy area	Sector
Domestic (*n* = 39) International (*n* = 4)	Drug control (*n* = 13) Public health (*n* = 20) Agriculture (*n* = 6) Economy (*n* = 2) Law (*n* = 2)	Government (*n* = 17) Nongovernmental organization (*n* = 13) Intergovernmental organizations (*n* = 5) Academic (*n* = 14) Commercial (*n* = 3)

### Documentary analysis

The analysis draws principally on interview accounts, but also uses documents to construct an overall narrative of the policy process. Documents were primarily drawn from written and verbal submissions of individuals and organizations that participated in the parliamentary debate between 8 August 2012 and 13 December 2013. Official documents from Uruguay’s Parliament were retrieved through its searchable online database (Parliament of Uruguay, 2024) using the search terms ‘cannabis’ and ‘cannabis regulation and control’ [‘regulación y control de cannabis’ in Spanish].

Information from the initial document search was used to conduct more targeted searches using the search engines of the Office of the President (2024), the Institute for the Regulation and Control of Cannabis (IRCCA, 2024), the Drug Control Board (JND, 2020) and the ministries of health (MSP, 2024) and agriculture (MGAP, 2024). A total of 33 documents were analysed, including position papers, draft legislation, parliamentary meetings and consultation proceedings. News reports and information gathered from the websites of nongovernmental organizations identified as relevant were also analysed.

### Fieldwork observations

The fieldwork process was designed to be adaptable to the dynamics of the Uruguayan context and to respond to the possibility for data collection. This meant that the stories and experiences that emerged from data collection during the initial stages of fieldwork would inform later aspects of the research process. A considerable amount of time was spent in the field (12 months), which allowed the researcher to be immersed in the social and political context under which cannabis regulation was developed and to observe its complex nuances. While perceptions about the pursuit of policy coherence have been explored via interviews (Lencucha *et al*., 2016; Battams and Townsend, 2018; Baker *et al*., 2019), how context shapes and constrains the decisions of policymakers can be implicit and difficult to articulate. Complex policy processes are therefore best observed in real-life settings (Dwyer and Buckle, 2009).

Extensive fieldwork notes were taken following informal conversations with relevant policy actors and key events attended. Key events included the 2017 Uruguay Cannabis Expo (ExpoCannabis Uruguay), a knowledge exchange event held annually in Montevideo for cannabis users, the cannabis industry and regulators of the cannabis market (ExpoCannabis Uruguay, 2024) and the Cannabis Cup (Copa Cannábica), an annual event organized by the Uruguayan Association of Cannabis Studies (Asociación de Estudios Cannábicos del Uruguay) that brings together members of cannabis clubs and home cultivators in Uruguay (Capurro, 2016). During observations, interest was taken in understanding how contextual factors influenced the ways and extent to which policy actors drew on policy models for tobacco and alcohol and how they reflected on their relevance (e.g. normative or strategic) to cannabis regulation. Insights about the study context with respect to cultural norms (e.g. beliefs about the role of the state) and societal pressures (e.g. public concern about the risks of commercialization, high approval rating for tobacco control) were recorded and drawn on to understand the policy environment in which research participants were situated.

In line with Dwyer and Buckle (2009), fieldwork notes were also drawn on during reflective stages of the project to assess how the researcher’s point of access, identity, social position and subjectivity might influence the nature and conduct of this research (see below). Field observations were collected either through handwritten notes or dictation via an audio recorder. These were not shared or archived, and all files containing identifiable information about interview participants were destroyed at the end of the project.

### Thematic analysis

Interviews were analysed in NVivo 10 data analysis software using an iterative thematic approach, in which codes and sub-codes were developed inductively through repeated readings of the transcripts. In the first stage, interviews were scanned to identify instances where tobacco and/or alcohol experiences and approaches were drawn on to support policy positions. For the purposes of this study, relevant instances were defined as perceptions describing policy coherence with tobacco and/or alcohol in terms of (i) its primary use as normative or strategic, (ii) the type of horizontal coherence advocated as internal (within health) or external (across health and public security), (iii) and the objectives of policy (in)coherence (e.g. combat illicit market and prevent youth use). Views on the actual or perceived impacts of policy incoherence on health and other outcomes in the post-implementation stage were not included.

An integrative approach to thematic analysis was used and relevant themes identified within the interview data were applied to analyse documents and fieldwork observations, which were reviewed to identify any differences in terms of key themes and findings. This process allowed for the generation of themes that were consistent across the entire dataset, ensuring that a high level of convergence had been achieved (Farmer *et al*., 2006). Consequently, although interview data constitutes much of what is quoted in this article, it is representative of a wider dataset.

The researcher’s identity as an outsider to the Uruguayan context, non-native Spanish speaker and public health scholar may have influenced the nature of this research. On reflection, the researcher’s outsider status allowed them to build rapport with Uruguayan participants, and they appeared willing to elaborate on aspects of the local culture and historical context in detail. The researcher’s gender identity and status as a non-native Spanish speaker may have also influenced the nature of the discussions. For example, at times, some interviewees appeared surprised by the researcher’s ability to write and speak Spanish fluently, and younger male participants (aged 25–40) tended to dominate the conversation and discuss issues of personal interest; however, like older male participants, they were also willing to elaborate and explain their positions in more detail if requested. To ensure that the researcher’s positionality as a public health scholar did not bias interviewee responses, an ambiguous position on cannabis regulation was presented and interviewees were requested to express their concerns, priorities and preferred policy approaches in an open and impartial manner.

## RESULTS

The pursuit of policy coherence was identified by interviewees as relevant to Uruguay’s cannabis policy process, albeit in diverse and often competing ways. The following sections describe these diverse perceptions of coherence with illustrative examples and their relevance to influencing the development of cannabis regulation.

### Horizontal coherence with tobacco: legal, available but not actively promoted

The promotion of policy coherence across cannabis and tobacco appears to have been presented as the official position of the Uruguayan government, though the extent to which this was pursued as a normative commitment is less clear. When asked how they sought to regulate cannabis along similar lines to tobacco, one high-level official suggested: ‘*Remember that we […] had an important change with the regulation of tobacco, including we were world famous’* (Regulator). This reference to Uruguay’s international status as a leader in tobacco control can be similarly observed at a 2013 press conference following advancement of the cannabis regulation bill to legislative debate, where former Undersecretary Diego Cánepa asserted:

We are going towards strict regulation with cannabis, tobacco and alcohol. I think that there is enormous consistency […] Uruguay is an international leader in the anti-tobacco campaign and we have a strictly regulated market; the solution is not prohibition, it is to strictly regulate the cannabis market. (Cánepa, 2013)

To other key actors, the experience with tobacco was presented as demonstrating that through regulation, the state could more effectively prevent corporate control of the market. It was suggested that regulating cannabis along similar lines to tobacco illustrated the positive impacts that regulation could have by placing strict limitations on the market conduct of cannabis producers, as one interviewee articulated:

Uruguay’s tobacco policy established a regulated market, where, for example, advertising was prohibited, where youth use was cut in half, from 34% to 17%, and where it managed to increase risk perceptions for tobacco use through public education campaigns […] our tobacco policy generated the ‘know how’ that it was possible to regulate the drug market and have positive effects, limiting the actions of cannabis companies. (Politician)

By applying the tobacco model, this would have implied the use of education campaigns to discourage use at the population level (Abascal and Lorenzo, 2017); however, this did not materialize. Instead, the way that some interviewees presented the approach suggested that they felt use should be discouraged among youth but not the entire population. Conceptualized this way, applying the tobacco model to cannabis regulation implied that there would be an education campaign to discourage youth use and increase risk perceptions among the public with the overriding objective of preventing excessive consumption. It also meant that the state would intervene to prohibit marketing that promoted excessive use but, in general, use would be tolerated and not actively discouraged. Select aspects of the tobacco model that most participants felt were relevant for cannabis regulation therefore included the ‘*good lessons, for example, the issue of no marketing, no branding*’ (Civil society), thereby providing a blueprint for cannabis regulation to:

Reduce marketing as much as possible but for those that want to consume it can do so freely. What is restricted more than anything is marketing related to consumption. Prioritise information on the risks of consumption, but consumption is allowed. (Agriculture regulator)

Critics of this regulatory approach for cannabis argued that it was overly restrictive and stigmatized users. However, a small majority recognized the potential benefits of making links between cannabis regulation and Uruguay’s tobacco model in terms of increasing popular support for the reform. Interview accounts show that some reform advocates were exploiting the fact that ‘*tobacco regulation was widely accepted in Uruguay*’ (Civil society) to convince the public that use would not increase after liberalizing cannabis supply:

The tools given by regulation, to curb tobacco use without banning a single cigarette […] Honestly, I think it is one of the more convincing points that Uruguay has managed to use regulation in such an effective way to reduce tobacco use by 30%. (Politician)

The ambiguity surrounding whether cannabis would be regulated like tobacco was particularly concerning for those interested in regulating unhealthy commodities through a population health approach. Population health advocates were concerned by the potential impacts of liberalizing cannabis supply on tobacco denormalization goals, if it were to, for example, renormalize smoking. Accordingly, they called for an approach to cannabis regulation that was not only consistent with but mutually reinforced Uruguay’s tobacco control objectives. This implied that like tobacco, cannabis would be legal and available, but the state would be ‘*actively’* (Tobacco control lawyer) pursuing actions and programmes to discourage use at the population level. Aspects of the tobacco model that were perceived as relevant for cannabis regulation therefore included:

…that the government would develop adequate prevention and education campaigns to influence the population. That there was not going to be any kind of marketing. That it was not going to allow people to smoke in enclosed spaces and that it was going to respect the tobacco control law […] It seemed logical to homogenise the two policies: you can’t consume in enclosed spaces any type of smoked product. (Tobacco control advocate)

Concerns were raised by health advocates around the ambiguity regarding whether a goal of cannabis regulation was to prevent population-level use or reduce use-related harms for current users. Uruguayan officials had little apparent interest in applying measures that went beyond persuading individuals to consume in responsible ways, which suggests they were more aligned with the second aim. Indeed, there was general resistance by proponents of reform to the idea that cannabis should be regulated to the same degree as tobacco based on the claim that each substance requires a framework that is specific to its unique historical and legal context, consumption culture and relative risks and harms of use.

### Horizontal coherence with alcohol: promoting responsible use

While high-level officials publicly claimed a deliberate effort was made to promote consistent regulation across cannabis and alcohol, it is not entirely clear how they sought to align policy goals and instruments across the two health issues. In the interviews, participants were therefore requested to elaborate further on this claim by explaining the ways in which they would regulate cannabis along similar lines to alcohol. In response, one key official asserted:

I think that something has been left out and mostly hidden behind the reason for why we did this, which is that I was a strong proponent of regulating alcohol use in Uruguay […] The idea was to bring both to a middle point and strongly regulate alcohol and cannabis. (Regulator)

When other participants were asked this question, most were surprised by the reference to alcohol. Instead, interview accounts suggest that a key goal for some participants was to avoid the development of a market like alcohol, in which private interests seek to maximize profit by promoting excessive consumption and minimal regulation. In this context, Uruguay’s alcohol model was often presented as providing ‘*important lessons of what not to do*’ (Civil society) in the case of regulating cannabis. One interviewee even suggested that there was broad consensus regarding how ‘*it was clear that the cannabis market would not be dominated by the industry like alcohol*’ adding that ‘*we do not want that market for the adult use of cannabis nor for alcohol, where the industry created the need to consume and was part of the problem’* (Physician).

For most participants, the alcohol experience demonstrated that unregulated corporate control of the market might enable the development of an upstream driver that would seek to increase consumption through marketing campaigns aimed at young people. A decade of changing alcohol use patterns in Uruguay was presented as a key example of these adverse consequences and their implications for a legal cannabis industry. As one regulator argued:

Over the last 10 years, a campaign aimed at young people changed consumption patterns and that produced a cultural change of important magnitudes in our society. Between that period, there was a concentration of the beer industry in Uruguay, Argentina and Brazil, as there was worldwide. (Drug regulator)

Interview accounts suggest that policymakers also drew on Uruguay’s experiences with alcohol to inform an approach to cannabis that sought to avoid the development of a structurally powerful force that would seek to exert political influence over the policy process. It was suggested that without state control over market conduct, this would create structural barriers to passing cannabis regulation in the future, as political pressure to promote minimal regulation would source from diverse sectors of the industry:

I think the principal thing is that authority remains under state control because we run a huge risk like what occurs with alcohol today where half the population consume it daily in Uruguay. There are strong economic interests, from the workers to the companies that make it difficult to pass regulation. Because each time someone wants to regulate the alcohol market, the beer companies, wine producers and whiskey importers jump up. (Politician)

The view that the marketing and political practices of the alcohol industry were the key underlying drivers of alcohol-related harm in Uruguay did pose some challenges for proponents of reform. Although wanting to avoid a commercial cannabis market, an important aspect of Uruguay’s alcohol model that some key actors found more appealing than tobacco was the goal of preventing excessive consumption. Underpinning this policy preference was the belief that substance abuse affects a minority of people who consume alcohol and cannabis in harmful or irresponsible ways. However, in contrast to understandings about tobacco-related harm, most participants felt that ‘*if you moderate your alcohol consumption, you don’t have a problem. There is a safety threshold with alcohol that you don’t have with tobacco. Only one cigarette harms you’* (Drug regulator).

Consequently, Uruguay’s alcohol model has never sought to eliminate use entirely but rather to promote responsible use for adults and discourage use in children and pregnant women. These were central aspects of the alcohol model that some proponents wanted to bring to cannabis regulation, as they were seen as consistent with the goal to ‘*guarantee the liberty to consume and restrict promotions on consumption that drive people to consume excessively’* (Drug regulator). Promoting responsible use seemed like the most appropriate approach for the cannabis education campaign, as one interviewee explained:

The campaign should have about the same characteristics as the alcohol campaign, it should deal with promoting responsible use. (Physician)

The shared goal of promoting responsible use to respect the liberty to consume within certain parameters led to policy coherence across alcohol and cannabis in terms of who may purchase, possess or consume cannabis and what activities may not be associated with consumption. For example, there was broad support for a prohibition on sales to minors under 18, despite recognition that age restrictions rarely prevent youth access to either tobacco or alcohol:

The alcohol law prohibits sales to minors under 18 years old. So, are we going to allow cannabis sales to minors? That would be inconsistent. Tobacco cannot be sold to minors […] There would be an incoherence from the get-go if we were to permit legal sale and access to minors of cannabis. (Tobacco control advocate)

In terms of what activities may be associated with consumption, ‘responsible use’ was taken to mean that like alcohol, cannabis should not be permitted while driving or operating heavy machinery. This meant that the zero-tolerance policy required for alcohol should be applied to cannabis as the two substances were viewed as chemically similar relative to tobacco.

These restrictions were deemed reasonable as they guaranteed the liberty to consume for adults while enforcing personal responsibility to consume cannabis in ways that did not harm bystanders, as one interviewee put it:

I cannot drive if I am under the influence of cannabis. It seems to me that you have a social responsibility to say well, I like to drink alcohol, one drink at home […] But that cannot be during working hours or while driving. Those types of regulations should exist. (Academic)

### Horizontal coherence across health and public security agendas

This section presents the case of policy incoherence in the debate around efforts to reconcile tensions across public security and unhealthy commodity regulation policies focusing on ideational conflicts, and policies regulating the price and availability of cannabis.

#### Ideational conflicts: neoliberalism versus public health

Interview accounts identified fundamentally different values between public security proponents and population health advocates, which seem to stand in the way of each other’s objectives. Population health objectives seek to make unhealthy commodities less desirable and attractive through: (i) increased prices and (ii) restrictions on accessibility and availability. However, if this approach were applied to cannabis regulation, then it was seen by public security advocates as likely to undermine the state’s capacity to capture the illicit market from organized crime by making that market more attractive to consumers. Although public security advocates were not firm believers in neoliberal ideology, they did feel that in a free market system, if the state did not facilitate the flow of and access of cannabis, then it may lose potential clients to more competitive drug traffickers:

It’s all from the point of view of a liberal economy, which is something very questionable. But, from a strategic point of view, to be able to commit to developing this policy, I think that it was key. (Politician)

In contrast to population health advocates, achieving the goals of public security required a strategy that made the legal market more attractive and desirable through three interrelated ways: (i) reduced prices; (ii) better-quality products; and (iii) increased accessibility and availability. Underpinning this position is a neoliberal assumption that the role of the state is to facilitate market competition, which contrasts sharply with population health objectives to strengthen state capacity for intervening in and regulating unhealthy commodity markets (Battams and Townsend, 2018). In legislative debate, politicians and population health advocates asserted that competing with the illicit market by encouraging users to switch to the legal market had implications for the state’s capacity to engage in a serious effort to inform the public on the harms of cannabis use. Instead, the neoliberal approach underlying the public security strategy was seen as posing the serious consequence of:

Sending the message that cannabis use is without risk, which could normalise consumption. As something banal that would become more accessible and consumption would expand. (Tobacco control advocate)

#### Price policy

The public security objective gained prominence in the policy formulation stage, such that it led to a price policy for cannabis that conflicts with the objectives of unhealthy commodity regulation. Although some government actors with public security concerns were willing to apply high prices to tobacco and alcohol to reduce consumption at the population level, they were reluctant to treat cannabis in the same way. Rather, they felt that if the proposed cannabis regulation bill were to follow that strategy, then it would discourage users from purchasing cannabis in the legal market because the price would exceed the cost of cannabis in the illicit market, thereby making that market more attractive:

The objective of the [price] policy was to provide a substance that people were consuming in an illegal way, transforming that consumption into something legal. So, with respect to tobacco and alcohol, which are substances that are consumed legally, it does not seem bad to me to make them less attractive through higher taxes. (Agriculture regulator)

A potential reason for this limited consistency across public security and unhealthy commodity regulation goals was the lack of involvement by prominent health authorities in Uruguay to promote a price policy for cannabis that aligned with Uruguay’s approach to regulating unhealthy commodities, particularly tobacco. As one tobacco control advocate explained:

There was discussion that if we put the price too high, then the users are not going to buy cannabis from the state because clandestine cannabis will be cheaper. That is an issue that we did not get into that much. It was not our objective to discuss how much the price would be. (Tobacco control advocate)

This situation may have been exacerbated by the strategic appropriation of public security arguments by harm reduction and civil liberty advocates to the effect of promoting a price policy for cannabis that was inconsistent with the goals of Uruguay’s tobacco model. One harm reduction advocate declared that Uruguay should be:

Cautious with the tax issue. We cannot forget that this policy also has an objective to combat and steal the illegal market from criminal organisations, take from them the largest part of the market. […] We have to compete in price. (Physician)

#### Availability and accessibility

The liberalization of the cannabis supply chain was seen by those with public security concerns as a central strategy for increasing the quality and quantity of legal cannabis while combating the illicit market. Providing legal access to cannabis through multiple supply channels was presented as an integral part of that strategy. Public security advocates argued that if the state only legalized part of the market through home cultivation or cannabis clubs, then this would pose the risk of stimulating growth of the illicit market, as public demand for cannabis would not be satisfied.

For population health advocates, facilitating access to cannabis raises tensions with the objectives of tobacco and alcohol control, as both seek to increase barriers to access to reduce consumption. Moreover, some population health advocates were concerned that increasing the legal supply of cannabis through multiple channels posed the risk of exposing youth and nonusers to cannabis, as it would be widely available in the community. Contesting the neoliberal approach underlying the public security strategy of Uruguay’s cannabis regulation, one politician argued:

Through competition, there will be more availability of cannabis in the community and thus it will be more accessible. It will be easier for minors to access and use it, even if they are not allowed to purchase it. (Politician)

## DISCUSSION

The analysis demonstrates that policy actors selectively drew on policies and experiences from the regulation of tobacco and alcohol in ways that promoted particular features while discarding others. The promotion of policy coherence within health seemingly reflects a desire by policymakers to develop cannabis regulation that could achieve the implicit goal of legitimating cannabis use without creating an upstream driver or structural force that would promote excessive consumption.

**Fig. 2:**
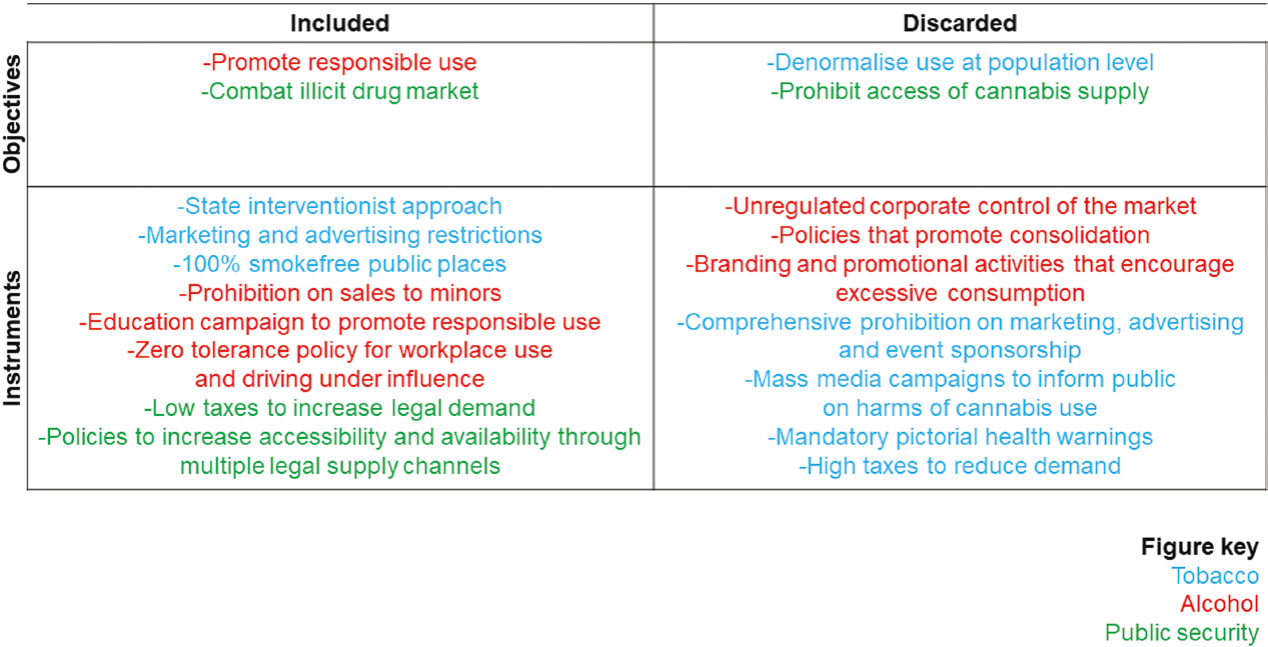
Extent to which concerns for policy coherence across health and public security policies shaped the outcome of Uruguay’s cannabis regulation.

The findings also illustrate the considerable substantive and political obstacles of reconciling tensions between unhealthy commodity regulation and public security objectives. Policy actors discarded policies and experiences from tobacco and alcohol control, particularly those elements that would reduce demand for the legal market (Figure 2). In turn, the policies and practices presented as driven by public security concerns are those that raise tensions with unhealthy commodity regulation goals, including interventions that stimulate demand for the legal cannabis market rather than alter levels of consumption among the population. This result may be explained by concerns expressed by some public security advocates that if the state did not facilitate the flow of and access to cannabis, then it may lose potential clients to more competitive drug traffickers.

The findings qualify and challenge claims made by some key officials in Uruguay that cannabis regulation constituted part of a consistent strategy to regulate unhealthy commodities, namely tobacco and alcohol, based on the state’s wider commitments to public health and rights-based approaches (Cánepa, 2013; Romani, 2015). The analysis demonstrates that ideas from these experiences about the importance of preventing widespread marketing campaigns and unregulated corporate control of the market found in prior research (Arocena and Aguiar, 2017; Forné, 2017; Musto, 2018; Rivera-Vélez, 2018) are evident within the policy to some extent.

However, the process of coordinating ideas about the CDoH appears to have been pursued in ways that were consistent with or subordinated to the public security objectives of cannabis regulation. The data indicate that public security advocates did not seem particularly concerned by the risk that cannabis use may increase as an outcome of policy change, as the overriding objective to combat the illicit market was seen as more important. This underlying premise seems to suggest a situation in which Uruguay’s public security policy is removed from the practices and goals of unhealthy commodity regulation. In this context, it is not surprising that population-based measures that target the upstream drivers of ‘ill health’ (such as those relating to demand reduction) have encountered significant barriers in the development of Uruguay’s cannabis regulation.

This study has some limitations, including those inherent to single case studies (Grandy, 2009). Focus on the pursuit of horizontal coherence within government may risk neglecting the significance of vertical coherence challenges across Uruguay and its international drug and health obligations. However, this emphasis reflects evidence provided by interviewees, highlights an emerging source of policy incoherence in this field and contributes to the public health literature on policy coherence that focuses primarily on challenges to reconcile tensions across trade and health agendas.

This research highlights the need to expand and deepen understanding of the conditions that foster policy incoherence across health and other policy spheres. Elsewhere, public health scholars widely argue that efforts to establish coherent approaches to regulating the CDoH are challenged by the political activities of transnational corporations (TNCs) (Ulucanlar *et al*., 2023), notably producers of unhealthy commodities, including tobacco (Collin, 2012), alcohol (Hawkins *et al*., 2018), ultra-processed food and drinks (Lauber *et al*., 2021) and gambling (van Schalkwyk *et al*., 2024). This research suggests that TNCs engage in a diverse range of practices that serve to prioritize profit over public health (Gilmore *et al*., 2023). However, insights gathered from the current article indicate that health policy scholars would have much to gain by re-examining this assumption given the comparative absence of corporate actors in the case of cannabis in Uruguay.

For instance, Lencucha and Thow (2019) identify the potential significance of paradigmatic and institutional contexts in influencing the underlying conditions that shape policy environments that prioritize economic development over health protection goals. Future research might therefore explore the underlying conditions relevant to explaining why policymakers continue to promote inconsistent policies between health and other spheres because of competing national priorities. More broadly, research is critically needed to understand how the cannabis industry engages with and influences framing that leads to potentially different types of regulatory approaches across diverse contexts, particularly as more governments move to legalize and regulate recreational cannabis markets.

### Implications

One element of this research that is of potential significance to the development of a more coherent approach to regulating the CDoH is the evidence of potential synergies that the public security agenda has created with other policy sectors beyond health. The neoliberal assumption underpinning Uruguay’s public security strategy has arguably created an opportunity for policy coherence across relevant economic sectors, which have comparable priorities of reduced tariffs and market expansion. Unhealthy commodity regulation advocates should be cautioned by the interactions that have been created across these other policy spheres that have a bearing on health policy, particularly as more jurisdictions liberalize recreational cannabis supply. As is the case with conflicts between trade and health (Shaffer *et al*., 2005; Collin, 2012; Schram, 2018), these synergies could present further challenges for policy coherence across health issues if population health advocates have to compete with the overriding objective of combating the illicit market alongside economic development goals.

This study also raises questions around whether policymakers should treat policy coherence as a normative goal. In the health literature, policy coherence is often discussed in aspirational terms, with an implicit expectation that activities in economic and trade sectors should align with health protection goals (Lencucha and Thow, 2020). However, the pursuit of policy coherence across health and other policies might not be feasible or even desirable because of other national priorities (Duraiappah and Bhardwaj, 2007), despite recognition of its adverse effects on other policy areas (Horký, 2010).

It is apparent that health is one among many concerns shaping policy decisions. Policymakers might require a more nuanced approach to regulating cross-cutting issues depending on the policy area, which may result in tensions across policy spheres, as is the case with trade ministries providing subsidies to unhealthy commodity producers while health departments work to reduce the availability of unhealthy commodities (Lencucha and Thow, 2019). Policymakers might also need to prioritize the goals of one sector over other policy aims, as observed in the Uruguayan case where public security advocates promoted a competitive price policy for cannabis, despite recognition that this may inadvertently increase population-level consumption.

## CONCLUSION

This analysis demonstrates that, despite claims made by policymakers in Uruguay, the pursuit of policy coherence within health governance is relatively limited and if there is an element of regulatory coherence, it is likely to be challenged by minimal levels of coordination in practice. While policymakers selectively coordinated elements from regulatory frameworks for tobacco and alcohol, the outcome of cannabis policy reform was more directly shaped by broader political considerations—including how to resolve tensions between public security and unhealthy commodity regulation goals. This study raises important questions around the extent to which Uruguay’s cannabis regulation was shaped by the explicit goal of policy coherence, suggesting rather that comparisons with tobacco and alcohol regulation were strategically used to justify the introduction of a legally regulated cannabis market.

## Supplementary Material

daae136_suppl_Supplementary_Appendix

## Data Availability

The data underlying this article cannot be shared publicly for the privacy of the individuals involved in the study. The data will be shared on reasonable request to the corresponding author.
