# Supplementary material for: Challenges achieving horizontal coherence across health and public security policies in formulating Uruguay’s cannabis regulation
Source: Health Promot Int. 2024 Nov 4;39(5):daae136. doi: 10.1093/heapro/daae136 (PMC11533143; doi:10.1093/heapro/daae136)
Supplement: daae136_suppl_Supplementary_Appendix [file daae136_suppl_supplementary_appendix.docx]

**Supplementary materials**

| **Appendix A. List provided by ‘gatekeeper’ in Uruguay** | |
| --- | --- |
| **Organisation** | **Role** |
| International Cannabis Corporation | Cannabis production company |
| Symbiosis | Cannabis production company |
| Montevideo Pharmacy Centre | Trade union representative |
| Montevideo Pharmacy Centre | Trade union representative |
| Pharmacy in Montevideo | Owner |
| Frente Amplio | Legislator |
| Monitor Cannabis | Researcher |
| Drug Control Board | Civil servant |
| Proderechos | Advocate |
| Cannabis Museum | Director |
| Cannabis Community of Uruguay | Member |
| Uruguayan Association for Cannabis Studies | Advocate |
| Cannabis Expo | Organiser |
| Interdisciplinary Group for Cannabis Studies | Researcher |
| Interdisciplinary Group for Cannabis Studies | Chemist |
| Uruguayan Medical Society | Physician |
| Uruguayan Society of Endocannabinology | Physician |
| Pharmacy Association of the Interior | Trade union representative |
| Batar Foundation | Advocate |
| Hemp Production Company | Owner |
